# Biomarkers in Invasive Pulmonary Fungal Infections: Where Do We Stand?

**DOI:** 10.3390/jof12020104

**Published:** 2026-02-01

**Authors:** Isabel Montesinos, Hector Rodriguez-Villalobos

**Affiliations:** Microbiology Department, Cliniques Universitaires Saint Luc, Université Catholique de Louvain, 1200 Brussels, Belgium; maria.montesinos@saintluc.uclouvain.be

**Keywords:** invasive pulmonary fungal infection, biomarkers, invasive pulmonary aspergillosis, Invasive pulmonary mucormycosis, pneumocystosis, new diagnostic tools

## Abstract

Invasive pulmonary fungal infections remain a major cause of morbidity and mortality among immunocompromised and critically ill patients. Rapid and accurate diagnosis is crucial for improving outcomes, yet conventional methods such as culture and histopathology suffer from limited sensitivity and slow turnaround times. Recently, significant progress has been made in the development and standardization of serological and molecular biomarkers that enhance the early detection of the key pulmonary fungal diseases, particularly invasive pulmonary aspergillosis and pneumocystosis. Diagnostic tools for mucormycosis, however, remain scarce. PCR tools have strong potential to significantly improve early detection, but they are not yet widely implemented, and standardized commercial assays remain limited. Accessible antigen-based tests with robust performance are highly anticipated and expected to become available soon. This review summarizes the current evidence regarding the optimal use of galactomannan, β-D-glucan and PCR-based assays, emphasizing how their performance varies according to the pathogen, the type of specimen and the host population. Specific challenges, such as differentiating colonization from infection in non-HIV *Pneumocystis* pneumonia or interpreting galactomannan and PCR in patients receiving mold-active prophylaxis, are highlighted. We also discuss how combining biomarkers can enhance diagnostic accuracy and support timely therapeutic decisions. A clear understanding of the strengths, limitations and appropriate interpretation of these diagnostic tools is crucial in an era of increasing host complexity, shifting fungal epidemiology, and expanding antifungal options.

## 1. Introduction

Invasive pulmonary fungal infections (IPFIs) in at-risk patients have gained major clinical relevance over recent decades, largely due to advances in medical practice such as the widespread use of immunosuppressive therapies, the increasing number of organ and stem cell transplants, and the application of invasive procedures in intensive care units. These infections are associated with high mortality rates, partly related to delayed diagnosis. Early diagnosis is therefore essential to improve the survival of these patients, who frequently present with multiple comorbidities. Additionally, the epidemiology of IPFIs is evolving, with an expanding and increasingly heterogeneous population at risk, including non-neutropenic intensive care unit (ICU) patients, individuals receiving novel targeted immunotherapies, patients with chronic lung diseases, and those with severe viral infections such as influenza or COVID-19, further complicating early recognition and diagnostic pathways [1,2,3,4,5,6,7].

Conventional laboratory methods, such as culture or histopathology, are time-consuming and have limited sensitivity, which restricts their utility as initial diagnostic tools for IPFIs. In this context, a range of serological and molecular biomarkers have been developed with the aim of improving diagnostic sensitivity and enabling earlier detection. However, no single biomarker provides perfect accuracy, and results must always be interpreted in conjunction with clinical and radiological findings, as well as host-related risk factors [8,9,10,11,12,13]. Several therapeutic strategies are used in the management of IPFIs, ranging from prophylaxis in high-risk patients to empirical therapy when clinical suspicion is high, but microbiological confirmation is lacking. The laboratory plays a central role in preemptive and targeted approaches, where biomarkers are applied for screening, enabling early intervention before overt clinical disease develops [9].

This review aims to summarize the current state of available biomarkers for the most frequent IPFIs, discussing their advantages and limitations, and to outline future perspectives in this rapidly evolving field. Emphasis will be placed on when and how these biomarkers can best be applied in clinical practice, whether for early diagnosis, risk stratification, or therapeutic monitoring. This topic is especially relevant in the current era, given the continuous evolution of treatment strategies and the introduction of new antifungal agents, where timely and accurate diagnosis is crucial to optimize patient outcomes.

## 2. Diagnostic Biomarkers in Invasive Pulmonary Aspergillosis

### 2.1. Clinical Context and Epidemiology

Invasive pulmonary aspergillosis (IPA) has long been recognized as a major invasive fungal infection in immunocompromised hosts, particularly patients with hematologic malignancies, hematopoietic stem cell or solid organ transplants [14]. However, in recent years, new risk groups have emerged, including patients with severe chronic obstructive pulmonary disease (COPD) exacerbations, advanced liver disease, or severe viral infections such as influenza or COVID-19. A common denominator among these groups is the use of high-dose corticosteroids and critical illness requiring ICU admission, often leading to delayed diagnosis due to the absence of classic host factors [2,3,15,16]. Influenza- and COVID-19–associated pulmonary aspergillosis (IAPA and CAPA) have emerged as major complications of severe viral pneumonia in ICU patients. These entities share similar mechanisms but display distinct clinical patterns, with IAPA usually occurring earlier and showing higher angioinvasive rates, whereas CAPA tends to develop later during ICU stay. Both conditions are associated with high mortality and may present as tracheobronchitis, a severe form often lacking specific radiological signs and particularly aggressive in transplant recipients [2,3,15,16].

Given these diagnostic challenges and the heterogeneity of at-risk populations, the use of mycological biomarkers has become essential to improve early detection and guide clinical decision-making in suspected IPA.

### 2.2. Galactomannan Detection

Galactomannan (GM) is a polysaccharide component of the *Aspergillus* cell wall that is released during hyphal growth and can be detected in blood or bronchoalveolar lavage (BAL) fluid several days (typically 5–8 days) before the onset of clinical or radiological manifestations. Since its introduction in the late 1990s, GM detection has become one of the cornerstone biomarkers for the early diagnosis of invasive aspergillosis and is included among the European Organization for Research and Treatment of Cancer/Mycoses Study Group Education and Research Consortium (EORTC/MSGERC) microbiological criteria [8,13,17,18,19].

Validated sample types include serum and BAL, while cerebrospinal fluid (CSF) GM testing, although not validated by most commercial kits, has proven helpful for the diagnosis of central nervous system (CNS) aspergillosis and is recognized within the EORTC/MSGERC criteria [13,20,21]. A recent systematic review and meta-analysis evaluating CSF galactomannan as a standalone diagnostic test for CNS aspergillosis reported moderate sensitivity but high specificity, supporting its use as evidence for probable CNS aspergillosis in patients with high pre-test probability, while emphasizing that a negative result cannot reliably exclude infection [22].

For many years, the Platelia *Aspergillus* (Bio-Rad Laboratories, Hercules, CA, USA) remained the reference assay, widely used and extensively validated for both serum and BAL testing [8,9,17,19,23,24,25,26,27,28,29,30,31]. Recently, the field has advanced with the introduction of additional commercial enzyme immunoassay (EIA) kits, chemiluminescent immunoassays (CLIA), and lateral flow-based methods, most designed to improve diagnostic performance and simplify laboratory workflow. CLIA platforms enable single-sample processing, full automation, shorter turnaround times, and lower risk of contamination compared to batch EIA systems [23,24,25,26,27]. Simultaneously, lateral flow tests have been developed as rapid, point-of-care options for GM detection. These tests deliver results within 30–60 min and can be performed directly on serum or BAL samples with minimal technical requirements. Their analytical performance is comparable to that of traditional GM assays, making them valuable tools in resource-limited settings or for bedside testing in ICU [28,29,30,31]. However, despite their speed and simplicity, a prudent interpretation of these new test results is essential. It requires further clinical evaluation before its incorporation into IPA diagnostic criteria and therapy decision-making.

The sensitivities and specificities of the different galactomannan detection methods are summarized in Table 1. The wide range of reported values across studies largely reflects differences in patient populations, underlying diseases, sample type, and antifungal exposure. In general, serum galactomannan shows better sensitivity in neutropenic patients, in whom angioinvasion promotes early antigen release into the bloodstream. In contrast, sensitivity decreases in non-neutropenic individuals or in those receiving anti-mold prophylaxis (AMP), in whom both fungal burden and pre-test probability are reduced. Testing on bronchoalveolar lavage (BAL) fluid remains the most sensitive approach in these groups [2,14,25,32,33,34].

In its most recent update, the EORTC/MSGERC has established higher galactomannan cut-off values than those traditionally used with the Platelia assay as mycological criteria for IPA. These thresholds differ from those recommended by the manufacturer, setting the positivity criteria at an optical density index (ODI) ≥ 1.0 in serum or plasma, BAL, or CSF, or at a serum ODI ≥ 0.7 with a BAL ODI ≥ 0.8 from the same patient. These revised cut-offs aim to increase diagnostic specificity while incurring only a minor reduction in sensitivity, and to promote greater standardization of IPA case definitions across clinical and research settings. However, they should be used with caution in patients receiving mold-active antifungal therapy or AMP, as antigen levels may be suppressed, potentially reducing test sensitivity in these populations.

Several authors have demonstrated that high initial serum and/or BAL GM levels are associated with poor clinical outcomes. Regarding GM kinetics, persistently elevated or non-declining serum GM levels after one week of treatment have been shown to predict therapeutic failure and increased mortality [52,53,54,55]. Building on these findings, Mercier et al. proposed an algorithmic approach that integrates serum GM kinetics into clinical management, based on baseline and Week 1 levels [53]. However, caution is needed in non-neutropenic patients and in those on AMP, as GM levels can fall due not only to antifungal therapy but also to hepatic/renal clearance and neutrophil activity, potentially leading to misleading trends [53,56].

In conclusion, in a preemptive strategy among hematologic patients during neutropenia, serum GM remains an effective screening tool. For non-neutropenic or critically ill patients, such as those with COPD, CAPA, or solid organ transplantation, BAL GM testing provides superior sensitivity and should be prioritized. Moreover, both baseline GM levels and their subsequent kinetics have demonstrated prognostic value, serving as potential biomarkers of treatment success and overall outcome in patients with invasive aspergillosis.

### 2.3. Aspergillus DNA Detection

Polymerase chain reaction (PCR) assays for *Aspergillus* DNA detection were included as a mycological criterion for IPA in the latest EORTC/MSGERC definitions. This inclusion has generated debate, as results across studies remain variable despite the standardization efforts promoted by the Fungal PCR Initiative and the growing number of commercial kits. In addition, the reclassification of cases from “possible” to “probable” IPA when galactomannan is negative, but PCR is positive, has contributed to ongoing discussion about its diagnostic value and interpretation [11,13,57,58,59,60,61].

PCR can be performed on serum, plasma, BAL, or even tissue biopsy samples, offering flexibility for both screening and confirmatory diagnosis, integrated into pre-emptive or diagnosis-driven strategies, respectively. Several commercial kits are now available, some integrating detection of azole-resistance mutations, while others combine detection of *Aspergillus* and Mucorales in a single assay. The performance of the different commercial assays appears to be broadly comparable [25,62,63,64,65].

Reported sensitivity and specificity values for *Aspergillus* PCR vary widely depending on sample type, clinical context, patient population, or AMP or treatment. Table 2 summarizes the main advantages and disadvantages of *Aspergillus* PCR in blood and BAL samples, along with performance data from a recent review of published meta-analyses by Cruciani et al. This review of meta-analyses highlights variability in reported sensitivities and specificities, reflecting heterogeneity in the studied populations and methodologies [66].

Similarly to GM, higher sensitivity has been observed in blood samples from neutropenic patients compared to non-neutropenic individuals [67]. Nevertheless, PCR in blood tends to become positive earlier than GM, particularly in plasma, which appears to be both more sensitive and earlier than serum [68,69]. The impact of AMP on PCR performance remains a matter of debate. Some studies have reported decreased sensitivity, while others have shown no significant effect [11,25,70]. In contrast to galactomannan, antifungal agents that disrupt the fungal cell wall may cause the release of nucleic acids, thereby maintaining, or even enhancing, the detectability of circulating fungal DNA. This mechanism could partly explain why PCR sensitivity is not clearly reduced under AMP. However, specificity may be affected for several reasons. First, AMP lowers the pre-test probability of invasive aspergillosis, which inherently decreases the positive predictive value of any test. Second, detecting fungal DNA from nonviable organisms may yield positive results in the absence of active infection. Yet, these findings may also represent very early or incipient infections that remain GM-negative due to antifungal exposure. Overall, distinguishing between these scenarios remains challenging and underscores the complex interpretation of PCR results in patients receiving AMP [11,57,70]. Specificity can be improved by requiring two consecutive positive PCR results, as recommended by the EORTC/MSGERC [13].

In contrast, BAL PCR shows similar performance in both neutropenic and non-neutropenic patients. Unlike blood PCR, it seems to be less affected by antifungal treatment or AMP, both in BAL and in samples from other infection sites [57,67]. Its diagnostic accuracy is comparable to that of GM, but, when using the appropriate assay, it provides the additional advantage of directly detecting azole resistance genes in clinical specimens. This is especially important because BAL cultures are often negative, and antifungal susceptibility testing cannot be performed [57,62,63,71,72]. Interpreting a positive *Aspergillus* PCR result in BAL fluid as the only biomarker should be done cautiously, since some authors suggest it may sometimes indicate colonization or contamination rather than true invasive disease. However, this idea is not universally accepted within the scientific community, since a positive PCR may also indicate an early stage of infection, before galactomannan detection. The EORTC/MSGERC’s recommendation of two positive BAL PCRs lacks clear guidance on whether tests should be duplicates, from different lung areas, or repeated over time, making implementation difficult [11,13,57,63,66,73]. In practice, the most reliable diagnostic approach is to combine BAL PCR with galactomannan testing or with concomitant serum biomarker assays, which significantly increases diagnostic confidence and specificity.

**Table 2:** Comparative performance and characteristics of *Aspergillus* PCR assays in blood and BAL samples.

Sample	Se (%)	Sp (%)	Main Advantages	Main Disadvantages
Blood	57–84 [66]	58–95 [66]	Detects *Aspergillus* DNA earlier than GM;Plasma may offer higher Se and earlier detection than serum [68,69]; Useful in high-risk neutropenic patients non receiving AMP [11]	Sensitivity reduced in non-neutropenic patients [67]; Sensitivity possibly decreased under AMP [11]; Positive results may persist after clinical resolution; Lower specificity in patients receiving AMP [57,70]
BAL	57–91 [66]	92–97 [66]	Similar performance in neutropenic and non-neutropenic patients; Maintains Se under AMP [57,67]; Comparable to BAL GM [72]	Invasive sampling procedure; Possible reduction in specificity due to airway colonization; Requires careful clinical and radiological correlation [11]

Abbreviations: Se = Sensitivity; Sp = Specificity; AMP = anti-mold prophylaxis; GM = galactomannan; BAL = bronchoalveolar lavage; EAPCRI = European *Aspergillus* PCR Initiative.

Overall, PCR-based detection offers a useful complement to antigen tests like galactomannan, improving diagnostic confidence when combined with clinical and radiological findings. When positive in both serum and BAL from the same patient, PCR has a strong predictive value for invasive disease, supporting its inclusion among the EORTC/MSGERC mycological criteria. In clinical practice, serum PCR is particularly valuable as a pre-emptive diagnostic tool in neutropenic patients without AMP, while BAL PCR is best suited for a diagnosis-driven approach in patients with compatible clinical and radiological findings.

## 3. Diagnostic Biomarkers in Invasive Pulmonary Mucormycosis

### 3.1. Clinical Context and Epidemiology

Invasive mucormycosis is a rapidly progressive and life-threatening fungal infection characterized by marked angioinvasion and extensive tissue necrosis. Although it typically affects immunocompromised patients, it can also occur in individuals with uncontrolled diabetes mellitus (DM), most often presenting as rhino-orbito-cerebral mucormycosis (ROCM), as well as in patients on prolonged corticosteroid therapy. During the COVID-19 pandemic, an alarming rise in mucormycosis cases was reported in India, especially of the ROCM form, often affecting patients with poorly managed diabetes mellitus and those on uncontrolled corticosteroid therapy [4,5]. Other clinical manifestations include pulmonary (associated with hematological malignancies and solid organ transplantation), cutaneous (typically post-traumatic or related to burns), gastrointestinal (rare, but linked to high mortality), and disseminated forms (common in immunocompromised patients and often fatal), reflecting the broad spectrum of disease depending on the portal of entry and host immune status [74,75,76,77].

*Rhizopus arrhizus* is the most isolated Mucorales species overall, especially in ROCM. However, the distribution of Mucorales species varies by geographic region and infection site. In Europe, *Lichtheimia corymbifera* is the second-most frequently isolated species, often associated with pulmonary infections in hematologic patients. In contrast, species such as *Apophysomyces* is rare in Europe but the second most reported in India, where they are frequently involved in cutaneous or post-traumatic forms [78,79,80].

In invasive pulmonary mucormycosis (IPM), the most common form among immunocompromised patients, clinical and radiological features often overlap with those of invasive aspergillosis, making differentiation difficult. However, distinguishing between these infections is critical, as antifungal treatments differ significantly: amphotericin B remains the first-line therapy for mucormycosis, while voriconazole, the treatment of choice for aspergillosis, is ineffective against Mucorales. Posaconazole and isavuconazole have activity against particular species, with better efficacy against *Rhizopus* and *Lichtheimia* and reduced susceptibility in *Mucor* spp. [76,81].

Early initiation of appropriate antifungal therapy is crucial for improving patient survival, but diagnostic tools for mucormycosis remain limited and challenging. Histology and direct microscopy remain essential tools when mucormycosis is suspected, as they help distinguish the broad, ribbon-like, sparsely septate hyphae of the Mucorales from the thinner, regularly septate hyphae of *Aspergillus*, thereby guiding appropriate antifungal therapy. Unfortunately, their sensitivity and specificity are limited. Culture sensitivity is even lower because the fragile hyphae of the Mucorales are easily damaged during sample processing, leading to frequent false-negative results. Nevertheless, culture remains important whenever possible, as it allows species-level identification and antifungal susceptibility testing, which are crucial for optimizing treatment.

There are no commercial serological tests available, and beta-D-glucan (BDG) assays or GM are not useful for this group of fungi. Recent advances in molecular techniques, especially PCR-based assays, are providing new insights into mucormycosis diagnosis, enabling detection in various clinical samples, including tissue biopsies, respiratory specimens, and blood [76,77,82,83,84].

### 3.2. Mucorales DNA Detection

Molecular assays have significantly enhanced the diagnosis of invasive pulmonary mucormycosis, especially when used on BAL or tissue samples from the infection site, where histopathological and cultural methods often lack sensitivity. Since its introduction in France in 2015, the use of Mucorales PCR has greatly enhanced diagnostic accuracy and helped improve survival rates, particularly among ICU patients [78].

In a recent meta-analysis, BAL PCR showed the highest sensitivity (97%) for the diagnosis of IPM, making it a valuable diagnostic tool for immunocompromised patients with suspected disease [85]. PCR performed on fresh biopsy material offers higher sensitivity than when using paraffin-embedded samples (86% vs. 73%); however, the latter remains valuable for species identification in cases with positive histology but negative culture, thus supporting early targeted antifungal therapy. Specificity seems similar across various sample types (95%). However, false-positive results can occur when testing samples from potentially colonized or contaminated sites, especially from the respiratory tract. In such cases, evaluating the fungal load along with the clinical and radiological context is crucial for accurately interpreting a positive PCR result and establishing a reliable diagnosis [85,86,87].

Because of the aggressive angioinvasion and early hematogenous spread of Mucorales, DNA can also be detected in serum, offering a minimally invasive diagnostic option, especially useful for patients in whom BAL or tissue biopsy is not feasible. Interestingly, one study showed that the circulating DNA load in mucormycosis is 10–100 times higher than in aspergillosis, which may explain the good performance of serum PCR in this context [88]. Nevertheless, blood PCR generally has lower sensitivity than PCR performed on BAL or tissue biopsy specimens (81%), although its specificity remains high (95%) [85]. Recent studies have shown that serum PCR can become positive approximately 4–9 days before histopathological or culture confirmation and 1–2 days before radiological evidence of infection [88,89]. Persistent PCR positivity has been linked to a 100% six-month mortality rate, whereas PCR negativity after seven days of antifungal therapy is associated with better outcomes [89]. Implementing twice-weekly serial PCR testing as part of a pre-emptive strategy can improve early diagnosis, an approach shown to enhance survival rates and enable monitoring of therapeutic response [85,89,90,91].

Early Mucorales PCR assays were mainly in-house, causing high variability in DNA extraction and gene targets and limiting standardization and comparison. European harmonization efforts improved consistency, and recent meta-analyses show comparable performance across assay types and specimen sources [85,89,91]. Quantitative PCR (qPCR) is generally preferred over conventional PCR due to its faster turnaround time, lower risk of contamination, and greater analytical sensitivity [85]. Several commercial PCR assays are now available, allowing reliable detection of Mucorales DNA in serum, BAL, or tissue samples [12,65,87,92,93]. Coinfection with *Aspergillus* and Mucorales can occur and should be suspected when invasive aspergillosis does not respond to voriconazole. PCR assays that detect both organisms simultaneously are especially useful in this situation [25,65]. On the other hand, current commercial PCR kits do not detect all Mucorales species, and the range of detectable genera varies between tests, with most not enabling species-level differentiation [12]. Additionally, there is significant potential for technical improvements in commercial tests, as evidenced by a recent qPCR standardization study where well-established in-house assays, optimized for extraction, elution volumes, and qPCR parameters, showed higher analytical sensitivity than several commercial kits [91].

There is growing anticipation that Mucorales PCR will be included as a mycological criterion in the upcoming revision of the EORTC/MSGERC definitions, with further progress expected from the development of new complementary biomarkers for diagnosing Mucormycosis.

## 4. Diagnostic Biomarkers in *Pneumocystis jirovecii* Pneumonia

### 4.1. Clinical Context and Epidemiology

*Pneumocystis jirovecii* pneumonia (PJP) remains a major opportunistic infection, but its epidemiology has markedly evolved. Thanks to the widespread use of antiretroviral therapy, the incidence of PJP in people living with HIV has declined substantially, while most current cases now occur in non-HIV immunocompromised patients. This heterogeneous group includes patients with hematologic malignancies, solid tumors, organ or stem-cell transplant recipients, and individuals receiving high-dose or prolonged corticosteroids or other immunosuppressive therapies [6]. Patients with HIV typically have a more insidious onset, higher fungal burdens, and respond better to treatment. Conversely, non-HIV patients often experience abrupt respiratory failure, rapid decline, and higher mortality despite lower fungal burdens. Recent studies have shown that mortality rates are particularly high in patients with metastatic solid tumors, chronic liver disease, and those receiving corticosteroid therapy, the latter being a common feature among many non-HIV PJP cases. Furthermore, the presence of multiple comorbidities and frequent concomitant conditions (such as bacterial coinfections or treatment-related lung injury), combined with the fact that many of these patients are not receiving prophylaxis, can obscure typical clinical and radiological manifestations, delay diagnosis, and further contribute to the increased mortality observed in this heterogeneous population [6,94,95,96,97].

From a diagnostic perspective, culture is not feasible and direct microscopic examination shows highly variable sensitivity (31–100%), generally higher in HIV patients and more effective when using immunofluorescence [10,98,99,100]. Despite these limitations, microscopic visualization of *P. jirovecii* remains the mycological criterion required to define proven PJP according to the revised EORTC/MSGERC definitions [13,101]. Serological assays that measure IgG or IgM have shown inconsistent results and are not routinely used. LDH is often elevated, especially in HIV-infected individuals, but its limited specificity reduces its diagnostic usefulness [98,102,103,104]. Given these challenges, especially in non-HIV immunocompromised hosts in whom early diagnosis is crucial to improve outcomes, there is a growing need for high-performance, rapid, and, whenever possible, minimally invasive diagnostic tools [6]. In this context, PCR-based detection of *Pneumocystis* DNA, in combination with BDG assays, has become a key part of the diagnostic process. The following sections will discuss their performance characteristics, advantages, and limitations in both HIV and non-HIV populations.

### 4.2. Pneumocystis DNA Detection

PCR assays, significantly more sensitive than conventional microscopy, play a central role in the diagnosis of PJP. In the current EORTC/MSGERC definitions, PCR helps classify probable, not proven, PJP, mainly because standardization remains limited and clear interpretative criteria distinguishing infection from colonization are still lacking. Quantitative PCR (qPCR) has improved diagnostic accuracy by quantifying fungal load, which is crucial for interpretation across different specimen types and patient populations. Although several studies have proposed fungal-load thresholds to differentiate colonization from active infection, no universal cutoff exists. This lack of standardization remains one of the main challenges to fully integrating qPCR as a standalone diagnostic criterion [13,101].

A major challenge is the varied behavior of PJP in HIV and non-HIV patients. In HIV-infected individuals, the fungal burden in the lower respiratory tract is generally very high, making PCR positivity strongly predictive of true infection. In contrast, non-HIV immunocompromised patients typically carry much lower organism loads, which increases the risk of detecting colonization rather than active disease [100,105,106,107]. Clinical interpretation is therefore substantially more complex, and false assumptions of colonization may delay treatment in a group where early diagnosis is crucial. Notably, recent data indicate that even low-level detection of *Pneumocystis* in non-HIV patients, previously dismissed as colonization, may indicate an increased risk of subsequent PJP, and prophylaxis should therefore be considered in selected cases [98,108].

To address these challenges, expert groups increasingly recommend using two qPCR thresholds: a lower cut-off below which colonization is likely, and a higher cut-off strongly indicative of active infection, with an intermediate “gray zone” requiring clinical, radiological, and laboratory correlation [100,101,105,106,107,109,110]. Importantly, these thresholds must be established locally by each laboratory, as analytical performance varies significantly across platforms, the targeted gene can markedly influence quantitative results, and optimal cut-offs also depend on the characteristics of the population being tested, particularly in the highly heterogeneous non-HIV group, where underlying diseases, immunosuppressive regimens, and fungal burden differ widely [101,110].

BAL is the respiratory specimen with the highest diagnostic yield for PCR and is the most extensively studied sample. However, BAL is an invasive procedure that may not always be feasible for fragile patients, leading to the use of less invasive samples such as induced sputum (IS) or upper-respiratory specimens (URT). In recent meta-analysis, qPCR consistently showed superior performance to conventional PCR, with excellent pooled sensitivities for both BAL (~99%) and IS (~98%) in HIV and non-HIV patients. These high sensitivities indicate that a negative qPCR result in either specimen type can reliably exclude PJP in many cases. In contrast, IS generally shows lower specificity, likely because positive results may indicate colonization of the bronchial airways rather than true infection [102,111].

URT samples, like nasopharyngeal aspirates or oral washes, tend to have lower sensitivities, and this reduction is even more significant in non-HIV patients because they generally have lower fungal loads burden. Nevertheless, URT samples usually maintain reasonable specificity, and when they are positive, they are more likely to indicate a significant fungal burden in the lower respiratory tract [98,102,111,112,113]. Some studies suggest that in non-HIV patients, PCR performance varies among non-BAL respiratory samples due to their generally low fungal burden, and a negative PCR result from induced sputum alone may not reliably exclude PJP [98,114,115]. Blood-based PCR show variable sensitivity but excellent specificity [98,102,116,117]. Table 3 summarizes the diagnostic performance of PCR according to sample type.

### 4.3. Blood β-D-Glucan Detection

BDG is a pan-fungal polysaccharide component of the cell wall and is widely used as a non-specific biomarker for invasive fungal infections. It can be detected in serum or plasma, but not in infections caused by Mucorales, *Cryptococcus* spp. or *Blastomyces dermatitidis* [9,98,119,120]. In addition, some BDG assays operate in single-sample format, reducing turnaround time and enabling timely diagnosis even in low-throughput settings. Several studies have shown that their diagnostic performance is comparable to that of traditional batch-based methods [121,122,123]. Positive BDG results should always be interpreted cautiously due to their panfungal nature and the high rate of false positives results, which may arise from certain antibiotics, immunoglobulins, albumin infusions, hemodialysis filters, severe mucosal damage, bacterial infections, or sample interference (hemolysis or lipemia). For this reason, BDG positivity should be combined with organism-specific tests, and current recommendations advise obtaining two consecutive positive results to improve specificity [13,101,124].

For PJP, BDG has become a key mycological criterion in the revised EORTC/MSGERC definitions [13,101]. Several studies have shown that serum BDG detection may precede the onset of clinical and radiological signs by 1 to 10 days, providing an earlier diagnostic window; however, its sensitivity could be reduced in patients receiving prophylaxis [125,126,127]. A recent meta-analysis reported an overall sensitivity of 91% and specificity of 79%, with clearly higher sensitivity in Patients with HIV (94%) compared to non-HIV immunocompromised hosts (86%). In HIV patients, a negative BDG result reliably excludes PJP with about 95% certainty. In non-HIV patients, the negative predictive value depends strongly on pre-test probability: BDG is most useful for excluding PJP when clinical suspicion is low, whereas higher pre-test probability requires confirmation with additional tests (e.g., PCR) [125]. Notably, BDG levels are often significantly elevated in PJP, and several authors have suggested using higher diagnostic cut-offs than those provided by commercial manufacturers to enhance specificity [127,128]. However, BDG levels can also be influenced by comorbidities and age [127,129]. In one study, a BDG cut-off was proposed specifically for the non-HIV population to help distinguish *Pneumocystis* infection from colonization. The threshold performed well in qPCR-positive patients with infectious diseases, solid tumors (excluding lung cancer), autoimmune or inflammatory conditions, and hematological malignancies. However, its usefulness decreased in individuals with lung cancer, interstitial lung disease, or nephrotic syndrome, where BDG values tended to be lower and diagnostic discrimination less reliable [129]. These findings further emphasize the significant heterogeneity within the non-HIV immunocompromised population, where BDG performance may vary considerably depending on the underlying condition.

In summary, combining BDG and PCR significantly enhances diagnostic accuracy. Both qPCR and BDG need well-defined diagnostic thresholds to differentiate true infection from colonization, which is particularly challenging in the diverse non-HIV population. In patients infected with HIV, a high fungal load typically means that a negative BDG test and/or a negative qPCR from deep respiratory samples can reliably rule out infection PJP. In contrast, in non-HIV immunocompromised patients, negative BDG results in serum and/or negative qPCR results from non-BAL samples should be interpreted carefully and always in the context of pre-test probability of PJP. A combination of a positive PCR and elevated BDG significantly raises the probability of true infection, especially in non-HIV immunocompromised populations [119,125,130,131].

**Figure 1:**
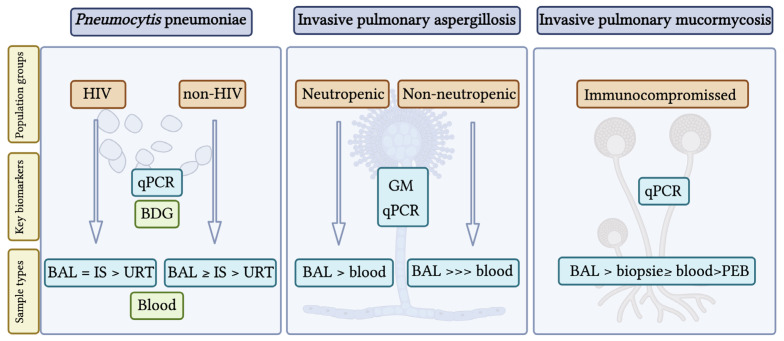
Overview of biomarkers in major invasive pulmonary fungal infections. Abbreviations: BAL = bronchoalveolar lavage; IS = induced sputum; URT = upper respiratory tract samples; GM = galactomannan; BDG = beta-D-glucan; PEB = paraffin-embedded samples; qPCR = quantitative PCR. Created in BioRender, https://BioRender.com/upwa45z (accessed on 3 December 2025).

Figure 1 presents a comprehensive summary of key biomarkers for invasive pulmonary fungal infections, highlighting how their effectiveness varies based on host factors and specimen type. This overview serves as a conceptual link between traditional diagnostics and the new tools discussed in the next section.

## 5. Other Invasive Pulmonary Fungal Infections

Pulmonary cryptococcosis can mimic a wide range of pulmonary infections as well as noninfectious conditions such as lung malignancies, making diagnosis particularly challenging [132,133,134,135]. Histopathology and culture remain important for definitive diagnosis; however, in routine laboratory practice, cryptococcal antigen (CrAg) detection is widely used because of its rapidity and ease of implementation [13]. In cases of isolated pulmonary cryptococcosis, serum CrAg testing appears to be less sensitive than in cryptococcal meningitis or disseminated disease, with higher sensitivity reported in patients with HIV compared with non-HIV immunocompromised hosts [134]. Several studies have nevertheless demonstrated good diagnostic performance of CrAg testing in bronchoalveolar lavage fluid, supporting its use as an adjunctive tool in pulmonary disease [134,136]. Molecular methods, including PCR, are well established for cerebrospinal fluid, although they generally show lower sensitivity than antigen detection. In pulmonary cryptococcosis, molecular assays applied to respiratory samples appear promising, but their clinical utility remains less well defined. Further studies are needed to clarify their role, as these methods are not yet widely implemented in routine diagnostic laboratories [134]. Importantly, the diagnosis of pulmonary cryptococcosis should prompt consideration of cerebrospinal fluid evaluation to exclude concomitant central nervous system involvement.

Dimorphic fungi, including *Histoplasma capsulatum*, *Talaromyces marneffei*, *Coccidioides* spp., and *Blastomyces dermatitidis*, are increasingly recognized causes of invasive pulmonary fungal infections, particularly in immunocompromised hosts and in the context of expanding geographic exposure. Pulmonary disease is the most frequent primary manifestation and is often clinically indistinguishable from bacterial or viral pneumonia, contributing to delayed diagnosis. Biomarkers play a central role in the diagnosis of pulmonary infections caused by dimorphic fungi. Their optimal use requires consideration of geographic exposure, host immune status, disease burden, and test-specific limitations, highlighting the need for integrated diagnostic algorithms rather than reliance on a single assay [137,138,139]. Immunological assays remain the mainstay of diagnosis for many dimorphic fungal infections. For histoplasmosis, antigen detection by EIA represents the cornerstone of diagnosis. *Histoplasma* antigen detection in urine and serum offers high sensitivity, particularly in disseminated disease and immunocompromised patients, although cross-reactivity with other dimorphic fungi may occur. *Histoplasma* galactomannan antigen LFA provide a rapid alternative with good performance in high-burden disease but lower sensitivity in localized pulmonary infection [137,140]. In talaromycosis, antigen-based assays targeting Mp1p antigens—including EIA, latex agglutination, and immunochromatographic tests—demonstrate high sensitivity and specificity, particularly in patients with HIV, and enable early diagnosis in endemic regions [138,141]. For coccidioidomycosis and blastomycosis, serological testing remains widely used, with enzyme immunoassays providing improved sensitivity compared with immunodiffusion or complement fixation, at the expense of reduced specificity due to cross-reactivity. Antigen detection in urine or serum is especially valuable in severe or disseminated disease [139]. Molecular methods, mainly in-house PCR assays targeting ITS regions or species-specific genes, have shown excellent diagnostic performance in disseminated infections caused by dimorphic fungi, particularly in immunocompromised hosts. However, sensitivity is lower in localized pulmonary disease, and lack of standardization and limited availability currently restrict widespread implementation in routine practice. These limitations reinforce the importance of multimodal diagnostic strategies combining serology, antigen detection, molecular testing, histopathology, and culture to ensure accurate diagnosis of pulmonary infections caused by dimorphic fungi [138,142,143,144,145,146].

## 6. New Diagnostic Approaches for Invasive Pulmonary Fungal Infections

Beyond conventional antigen and PCR-based assays, several new diagnostic technologies aim to improve the early and accurate detection of IPFI. A prototype lateral flow assay for Mucorales has demonstrated highly promising performance and could significantly assist in diagnosing pulmonary mucormycosis, especially when used in combination with targeted PCR. This type of assay would also enhance diagnostic capabilities in laboratories that have limited access to molecular biology platforms [147,148]. Antigen detection and PCR assays for less common respiratory fungal pathogens, like *Fusarium* or *Scedosporium*, show promising results in research settings; however, their current lack of commercial availability restricts their use in routine diagnostics [149,150,151]. Recent evidence suggests a potential role for fungus-specific IgM responses as adjunctive biomarkers in the early diagnosis of invasive fungal infections. Although still exploratory, IgM-based assays may complement antigen detection and molecular methods. However, their clinical interpretation remains challenging, particularly in distinguishing acute infection from chronic or latent colonization [152].

Pan-fungal PCR, followed by sequencing, allows direct detection and identification of fungal pathogens in clinical samples, although these assays currently remain in-house methods. A recent study highlighted the value of an integrated histo-molecular approach when fungal structures are visible on histological sections. This strategy demonstrated excellent accuracy in identifying genus and species levels, aiding more precise antifungal treatment, especially when culture and serological biomarkers are negative [153]. Molecular advances like digital PCR improve sensitivity and allow for earlier detection. Additionally, developing respiratory panels that include fungal pathogens is a promising future direction [110,154,155]. In parallel, metagenomic and targeted next-generation sequencing (mNGS and tNGS), as well as mycobiome profiling, offer broad, hypothesis-free detection of bacterial, fungal, and viral pathogens. Systematic reviews and meta-analyses have highlighted their promising diagnostic performance, particularly in complex cases where conventional methods are inconclusive. However, despite these encouraging results, their current clinical application remains limited by cost, technical complexity, and the need for further standardization and prospective validation [110,138,146,156,157,158,159,160,161]. Additional innovative strategies—including fungal volatilomics, proteomic and metabolomic signatures, and AI-assisted radiological analyses—are emerging but still require substantial validation [162,163,164,165]. Together, these evolving tools illustrate a shift toward multimodal and rapid diagnostic strategies that integrate antigen detection, molecular testing, and host-response markers to overcome the limitations of current assays.

## 7. Conclusions

Diagnosing invasive pulmonary fungal infections remains challenging due to the diversity of at-risk populations, variable clinical presentations, and the limitations of conventional microbiological methods. Biomarker performance varies significantly depending on host factors, fungal burden, and specimen type, highlighting the importance of careful interpretation based on the clinical context. No single assay achieves adequate accuracy across all scenarios. Integrating biomarkers into a multimodal diagnostic approach (combined with clinical evaluation, imaging, and microbiology) provides the most reliable path to early and appropriate antifungal therapy. Continued development and validation of accessible, rapid, and standardized assays will be essential for improving outcomes in these complex infections.

## Figures and Tables

**Table 1:** Comparative performance and characteristics of galactomannan detection methods in serum and BAL samples.

Method	Sample	Se (%)	Sp (%)	Main Advantages	Main Limitations
EIA	Serum	21–92 [35,36,37]	78–100 [35,36,37]	Widely validated; included in EORTC/MSGERC criteria [13] Good screening tool in neutropenic patients [2,14]	Batch testing; Long turnaroundtime; Reduced sensitivity under antifungal prophylaxis [32]; Cross-reactivity (β-lactams, other fungi, intravenous gluconate formulations, or cow’s milk in children) [38,39,40,41,42,43,44,45,46,47]
	BAL	35–89 [36,48]	79–100 [36,48]	Higher sensitivity than serum in non-neutropenic, ICU patients or patients receiving mold-active antifungals prophylaxis or treatment [2,14]	Requires bronchoscopy; Possible colonization findings
	CSF	69–81.1 [22]	94.4 [22]	Not validated by commercial kits but recognized by EORTC/MSGERC criteria [13]	
CLIA	Serum	11–100 [23,24,25,26,27,30,37,49]	65–100 [23,24,25,26,27,30,37,49]	Single-sample, fully automated, shorter TAT, reduced contamination risk	New assay-specific cut-offs require clinical and laboratory adaptation; Fewer validation studies: cut-off needs standardization
	BAL	52–100 [23,24,30,50]	65–100 [23,24,30,50]	Comparable to EIA; Flexible workflow	Interpretation thresholds still under evaluation
LFA/LFD	Serum/BAL	46–92 [51]	82–98 [51]	Rapid (<1 h); minimal equipment; suitable for POC or ICU settings	Lower analytical sensitivity. Subjective visual readout; not quantitative; Poor reproducibility [30]

Abbreviations: Se = Sensitivity; Sp = Specificity; EIA = enzyme immunoassay; CLIA = chemiluminescent immunoassay; LFA/LFD = lateral flow assay/device; BAL = bronchoalveolar lavage; CFS = cerebrospinal fluid; TAT = turnaround time; POC = point-of-care; ICU = Intensive Care Unit; EORTC/MSGERC = European Organization for Research and Treatment of Cancer/Mycoses Study Group Education and Research Consortium.

**Table 3:** Diagnostic performance of *Pneumocystis* PCR according to sample type.

Sample	Se (%)	Sp (%)	Key Considerations
BAL	98–99% [111]	85–95% [111]	Highest sensitivity: best for infection–colonization discrimination [98,111,115]; Invasive procedure; not always feasible in fragile or severely hypoxemic patients
IS	95–99% (HIV) [102,111]; 80–96% (non-HIV) [102,111,114]	80–98% [102,111]	Less invasive alternative; High sensitivity in HIV; potentially like BAL, although this is less consistent in non-HIV groups; specificity reduced by colonization [98,100,101,102,111,114,115,118]
URT	70–95% [102,111]	90–100% [102,111]	Simple collection; Lower sensitivity, particularly in non-HIV; a negative result does not exclude PJP; very good specificity [98,102,111,113]
Blood	Low–moderate	90–100% [102]	Minimally invasive; Low sensitivity, mainly positive in disseminated or high-burden disease (HIV patients); excellent specificity [98,102,116,117]

Abbreviations: Se = sensitivity; Sp = specificity; BAL = bronchoalveolar lavage; IS = induced sputum; URT = upper respiratory tract samples (nasopharyngeal aspirate, oral wash).

## Data Availability

No new data were created or analyzed in this study. Data sharing is not applicable to this article.

## Abbreviations

The following abbreviations are used in this manuscript:

EORTC/MSGERC	European Organization for Research and Treatment of Cancer/Mycoses Study Group Education and Research Consortium
IPFI	Invasive pulmonary fungal infection
ICU	Intensive care unit
IPA	Invasive pulmonary aspergillosis
SARI	Severe acute respiratory infections
IAPA	Invasive-associated pulmonary aspergillosis
CAPA	COVID-19-associated pulmonary aspergillosis
GM	Galactomannan
BAL	bronchoalveolar lavage
IS	Induced sputum
URT	Upper respiratory tract
CSF	cerebrospinal fluid
PEB	paraffin-embedded samples
CNS	central nervous system
EIA	Enzyme immunoassay
CLIA	chemiluminescent immunoassays
ODI	optical density index
LFA/LFD	lateral flow assay/device
TAT	turnaround time
POC	point-of-care
PCR	Polymerase chain reaction
AMP	anti-mold prophylaxis
DM	diabetes mellitus
ROCM	rhino-orbito-cerebral mucormycosis
IPM	invasive pulmonary mucormycosis
PJP	*Pneumocystis jirovecii* pneumonia
BDG	β-D-glucan detection
qPCR	Quantitative PCR
